# Assessing multi-target antiviral and antioxidant activities of natural compounds against SARS-CoV-2: an integrated in vitro and in silico study

**DOI:** 10.1186/s40643-024-00822-z

**Published:** 2024-11-27

**Authors:** Aisha Nawaf Al balawi, Jayda G. Eldiasty, Sahar Abd-El Razik Mosallam, Alaa R. El-Alosey, Alaa Elmetwalli

**Affiliations:** 1 Biology Department, University College of Haql, “University of Tabuk”, Tabuk, Saudi Arabia; 2 Women’s College for Arts, Science and Education, Ain Shams University, Cairo, Egypt; 3 Department of Mathematics, Faculty of Science, Tanta University, Tanta, 31527 Egypt; 4 Department of Clinical Trial Research Unit and Drug Discovery, Egyptian Liver Research Institute and Hospital (ELRIAH), Mansoura, Egypt; 5 Microbiology Division, Higher Technological Institute of Applied Health Sciences, Egyptian Liver Research Institute and Hospital (ELRIAH), Mansoura, Egypt

**Keywords:** Antiviral, Cytotoxicity, Propolis, SARS-CoV-2, Vero CCL-81

## Abstract

There is an urgent need for preventive and therapeutic drugs to effectively treat and prevent viral diseases from resurfacing as they emerge during the COVID-19 pandemic. This study aims to assess the antiviral effects of four natural compounds commonly used in traditional medicine to treat SARS-CoV-2 infection. A cytotoxicity, dose-dependent, and plaque reduction assay was performed on Vero CCL-81 cells to figure out their effects on the cells. Quantification of cytokines was assessed. In silico analysis for the selected compound was also evaluated. Results revealed that the compounds could disrupt the viral replication cycle through direct inhibition of the virus or immune system stimulation. The cytotoxicity assay results revealed that the compounds were well tolerated by the cells, indicating that the compounds were not toxic to the cells. This study evaluated the antioxidant capacities of propolis, curcumin, quercetin, and ginseng using ABTS, FRAP, and CUPRAC assays, revealing that propolis exhibited the highest antioxidant activity of ABTS with 1250.40 ± 17.10 μmol Trolox eq/g, with FRAP values reaching 1200.55 ± 15.90 μmol Fe^2^⁺ eq/g and CUPRAC values of 1150.80 ± 14.20 μmol Trolox eq/g at 1000 µg/mL, highlighting its potential as a potent natural antioxidant. The results of the plaque reduction assay revealed that the compounds could reduce the size and number of plaques, indicating that the compounds could inhibit the virus replication cycle. Subsequently, using molecular docking to analyze the effect of propolis, curcumin, quercetin, and ginseng as inhibitors, it was unveiled that the four compounds are likely to have the potential to inhibit the protease activity, spike protein S1, and RNA polymerase of SARS-CoV-2 and the virus titer was reduced by 100% after post-infection using propolis as an inhibitor control.

**Graphical Abstract:**

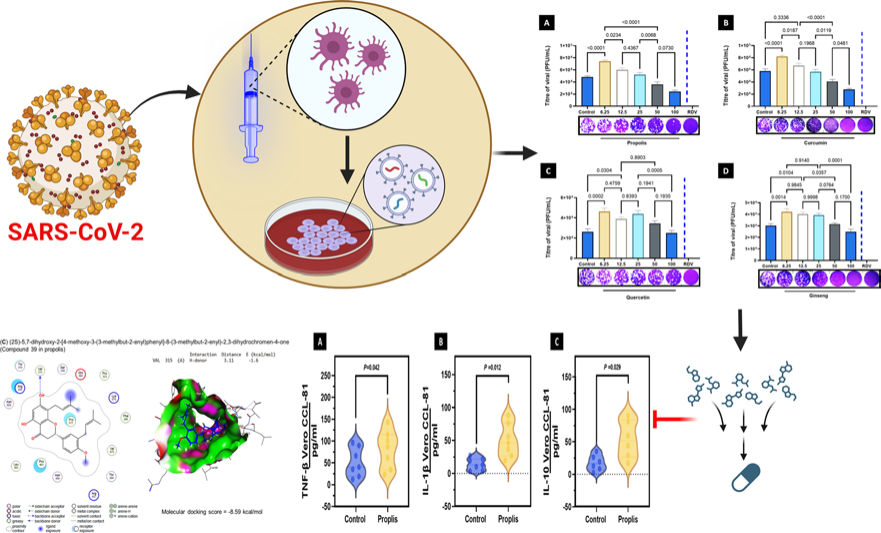

## Introduction

There is a continuing challenge for global public health in dealing with emerging and re-emerging diseases (Qiu et al. 2023; El-Alosey et al. 2023). Since 2003, several outbreaks have been caused by the Coronavirus family, which have addressed respiratory diseases or syndromes caused by these viruses (Gandhi et al. 2022).

In the course of history, humankind has faced numerous viral epidemics and pandemics, with devastating consequences for society and the economy (Ben-Nasr and Badraoui 2022; Badraoui et al. 2021; Ryan 2023). These viral outbreaks have disrupted daily life, leading to business closures, travel restrictions, and widespread social distancing measures. Moreover, the economic impact of such events can be severe, resulting in decreased consumer spending, job losses, and overall economic decline (Asif et al. 2022). There are seven different types of Coronaviruses in humans: SARS-CoV, MERS-CoV, and SARS-CoV-2 (Costanzo et al. 2022). These three viruses are the leading cause of severe acute respiratory syndrome in humans (Mousa et al. 2021), while the four most common human Coronaviruses, HCoV-HKU1, HCoV-OC43, HCoV-NL63, and HCoV-229E, are accountable for the majority of upper and lower respiratory tract infections among humans (Koryukov et al. 2022).

The use of vaccines is the primary means by which viral disease prevention is carried out, but antiviral drugs are also an essential complement, and they play an extremely crucial role in the prevention and control of viral diseases, especially during the lag time between the outbreak of a virus and the development of effective vaccines (Bakacs et al. 2022). Furthermore, when mutations occur among the circulating viruses, vaccines have the potential to lose their potency and become ineffective. Amidst the third Coronavirus outbreak in human history, the COVID-19 pandemic is one of the most urgent reminders of the urgent need for preventive and therapeutic drugs that can effectively treat and prevent the re-emergence of viral diseases as they emerge (Bardhan et al. 2023). For instance, the SARS-CoV-2 virus has so far been found to have mutated about 2% of the time, and these mutations could potentially affect the effectiveness of currently available vaccines (Araf et al. 2022).

The reality shows that it is imperative to develop practical therapeutic and pharmacologic approaches against the spreading of SARS-CoV-2 (Jiang et al. 2022). In addition, this scenario opens the door to examining the ancient practice of utilizing medicinal plants to treat diseases (Fornari Laurindo et al. 2023). This practice has been practiced by ethnic groups in Asia, the African continent, and South America for centuries (Jebaranjitham et al. 2022). This ancient practice could provide a new source of treatments with fewer side effects and more easily accessible to rural communities, where traditional healthcare is often limited (Desai and Mahitha 2022). Furthermore, medicinal plants could provide an effective first line of defense against the spread of the virus, as these plants could be used to prevent or treat symptoms before they become severe (Ben-Nasr and Badraoui 2022; Pawar et al. 2023; Badraoui et al. 2022).

In recent years, there has been a growing interest in using medicinal plants to mitigate various illnesses in developing nations, as they offer a viable alternative to primary health care (Howes et al. 2020). Numerous studies have demonstrated that medicinal plants contain active ingredients that can be found in modern prescription medications, and a significant portion of the world's population relies on natural medicinal compounds (Gunjan et al. 2015; Giannenas et al. 2020). Among the most commonly used medicinal plants are propolis (Hossain et al. 2022), curcumin (Subositi and Wahyono 2019), quercetin (Roy et al. 2022), and ginseng (Potenza et al. 2023), which have been employed in traditional medicines for centuries and possess diverse health properties (Table 1) (Salmerón-Manzano et al. 2020).

**Table 1:** Chemical structure and health-ornamental benefits of the selected compounds

Compound	Structure	Health-ornamental benefits	References
Flavone (commonly found in Propolis)		Anti-inflammatory, antioxidant, antiviral, antitumor, immunomodulation of pro-inflammatory cytokines	Braakhuis (2019)
Curcumin		Immunomodulatory, antiviral, and antiparasitic activity, synergistic interaction, anti-allergic activity, anticancer activity	Rathore et al. (2020)
Quercetin		Potent immunomodulatory, neuroprotective, psychostimulant, antiviral, antioxidant, anticarcinogenic, antimicrobial	Deepika (2022)
Ginsenoside		Improve vaccine efficacy as an adjuvant compound, enhancing innate and adaptive immunity, as well as inflammasome inhibitors	Peng et al. (2023)

As a whole, these natural compounds reduce and alleviate inflammation and are immunomodulating. The potential benefits of lowering lung damage, inflammation, and lung damage can be achieved through the reduction of nuclear factor kappa B (NF-_k_B), interferon beta- β (IFN-β), tumor necrosis factor- β (TNF-β), and other inflammatory factors (Cunha et al. 2022). There have been several studies that have demonstrated that plants rich in polyphenols may have potential therapeutic benefits in preventing the development of cytokine storms as well (Zinovkin and Grebenchikov 2020; Abou Baker 2022; Abbas et al. 2023). For instance, propolis has been extensively studied for its safety in various in vitro and in vivo models, with evidence showing low cytotoxicity at therapeutic doses (Sforcin 2016). Curcumin has a well-documented safety profile, with several studies demonstrating its low cytotoxicity in a wide range of cell lines, including cancer cells and non-cancerous cells (Rath et al. 2013; Joshi et al. 2021; Kah et al. 2023). Similarly, quercetin (Sartaj et al. 2021) and ginseng (Alsayari et al. 2021) have demonstrated minimal cytotoxicity in various studies, even at relatively high concentrations (Sun et al. 2022).

Therefore, this study aims to assess the antiviral action of four natural compounds most commonly used in traditional medicine in Tabuk, Saudi Arabia, for managing COVID-19 infection. Through cytotoxicity assays and plaque reduction assays, the compounds were tested to verify their antiviral activity against two strains of the SARS-CoV-2 virus, thereby validating their antiviral potential in the fight to combat SARS-CoV-2. Furthermore, the second goal, by testing the compounds using molecular docking, could be to figure out if the compounds can bind to the viral proteins and, if so, how tightly they bind; this will help to determine if the compounds have the potential to be developed into effective treatments for COVID-19.

## Materials and methods

### Materials

Our sources for propolis (CAS-No. 9009-62-5), quercetin (CAS-No. 117-39-5), curcumin (CAS-No. 458-37-7), and ginseng (CAS-No. 50647-08-0) were obtained in pure form (i.e., The study used individual compounds, considered "pure forms" rather than variable-concentration extracts) from Med Chem Express (Monmouth Junction, NJ, USA). A mixture of dimethyl sulfoxide (DMSO), 4,5-dimethylthiazoil-2-yl-2,5 diphenyltetrazolium bromide, and 3-(4,5-dimethylthiazoil-2-yl)-2,5 diphenyltetrazolium bromide from Sigma-Aldrich, USA (CAS: 298-93-1). Each compound was dissolved in 0.1 mL of DMSO and stored at 4 °C as stock solutions in 10 mM.

### Cell line and virus

We grew Vero CCL-81 cells (ATCC) were cultured in Dulbecco's Modified Eagle Medium (DMEM, Lonza, BioWhittaker^®^, USA) at 37 °C with 5% CO_2_, with 30% fetal bovine serum (FBS) (Lonza, BioWhittaker^®^, USA (CAS-No. 9014-81-7)), 1% penicillin–streptomycin (Lonza, BioWhittaker^®^, USA) (Cat. No. DE145-745E), and 2% heat-inactivated fetal bovine serum. Based on an Egyptian isolate BA.1 omicron strain, SARS-CoV-2 was used in infection experiments (Kandeel et al. 2023). A biosafety level 3 facility was used for viral culture at Haql College, Tabuk University, following laboratory biosafety guidelines (To et al. 2020).

### Cytotoxicity assay

An MTT (3-[4,5-dimethylthiazol-2-yl]-2,5 diphenyl tetrazolium bromide) assay was used to measure the effects of the selected compounds on Vero CCL-81 cells to ascertain the cytotoxicity of the compounds (Njeru and Muema 2021; El-Shehawy et al. 2023). To summarize, Vero CCL-81 cells were seeded in 96-well plates with 1.0 × 10^4^ cells per well in DMEM supplemented with 2% FBS at a density of 1.0 × 10^4^ cells per well. In the following step, the plates were incubated at 37 ºC with 5% CO_2_ for 24 h before they were collected. A dilution of 150 µL of compounds was added in quadruplicate to each well after the incubation, and the wells were kept at 37 °C with 5% CO_2_ for 48 h. After the PBS was added to each well, a final step was to add 30 µL of MTT, remove the supernatants, and wash with PBS twice. A 2 h incubation period was conducted after MTT addition, at 37 °C, using 5% CO_2_, and it was protected from light for 2 h. A 100 µL/well DMSO solution was added to the wells to dissolve the formazan crystals (El-Sewedy et al. 2023). Positive controls (cells treated with a known cytotoxic agent) and negative controls (untreated cells) were included in each experiment and were in triplicate.

### Dose-dependent assay

The compounds were analyzed using MTT assay data. By applying the Chou-Talalay method, the dose-dependent effect was calculated (Elwakeel et al. 2019). This calculation was performed using *CompuSyn* software. The dose–effect curves were created for both single treatments, and the fraction affected (Fa) values were determined for each dose and corresponding effect as previously reported (El-Sewedy et al. 2023). The dose-dependent assay was carried out in triplicate to guarantee reproducibility.

### Antioxidant ABTS scavenging activity

The activity of scavenging ABTS radical cation was assessed across various concentrations, and the outcomes were juxtaposed with those obtained from standard materials. The selected compounds and vitamin C were evaluated at corresponding concentrations, following the methodology outlined in a prior study (AlBalawi et al. 2023). Using the absorbance reading at 734 nm, the percentage of ABTS radical cation scavenging activity was computed using the formula:

$$ \% \,{\mathbf{ABTS}}\,{\mathbf{radical}}\,{\mathbf{cation}}\,{\mathbf{scavenging}}\,{\mathbf{activity}}\, = \,\left[ {{\mathbf{1}}\, - \,\left( {{{{\mathbf{A}}_{{{\mathbf{sample}}}} } \mathord{\left/ {\vphantom {{{\mathbf{A}}_{{{\mathbf{sample}}}} } {{\mathbf{A}}_{{{\mathbf{control}}}} }}} \right. \kern-0pt} {{\mathbf{A}}_{{{\mathbf{control}}}} }}} \right)} \right]\, \times \,{\mathbf{100Top}}\,{\mathbf{of}}\,{\mathbf{Form}} $$

### Cupric ion reducing antioxidant capacity assay (CUPRAC)

The CUPRAC assay evaluates the ability of antioxidants to reduce cupric ions (Cu^2^⁺) to cuprous ions (Cu^1^⁺), forming a stable colored complex with neocuproine. For the assay, a CUPRAC reagent is prepared by mixing 10 mL of 10 mM copper(II) chloride (CuCl₂), 10 mL of 7.5 mM neocuproine, and 10 mL of ammonium acetate buffer (pH 7.0). The samples were prepared in methanol at concentrations of 100, 250, 500, 750, and 1000 µg/mL. Trolox is again used as the positive control and prepared at the same concentrations. For the assay, 1 mL of each sample concentration or Trolox is mixed with 1 mL of CUPRAC reagent in a 96-well plate or test tube. The reaction mixture is incubated at room temperature for 30 min to allow a complete reduction of Cu^2^⁺ to Cu^1^⁺. The absorbance is then measured at 450 nm using a UV–visible spectrophotometer. The antioxidant capacity is determined by comparing the absorbance to a Trolox standard curve, and the results were expressed as µmol Trolox equivalents/g of the sample. All measurements are performed in triplicate.

### Ferric reducing antioxidant power assay (FRAP)

The FRAP assay is based on the reduction of ferric ions (Fe^3^⁺) to ferrous ions (Fe^2^⁺) in the presence of antioxidants. The reduced Fe^2^⁺ forms a blue-colored complex with 2,4,6-tripyridyl-s-triazine (TPTZ), and the intensity of the color is directly proportional to the reducing power of the sample, which can be quantified spectrophotometrically. For the assay, an acetate buffer (300 mM, pH 3.6) is prepared by dissolving 16.8 g of sodium acetate and 3.1 mL of acetic acid in 1 L of distilled water. The TPTZ solution (10 mM) is dissolved in 40 mM HCl, and the ferric chloride solution (FeCl₃, 20 mM) is prepared separately. The FRAP reagent is freshly prepared before the assay by mixing 10 parts acetate buffer, 1 part TPTZ solution, and 1 part FeCl₃ solution. This reagent is kept at 37 °C to ensure its stability during the reaction. For the sample preparation, different concentrations of samples were dissolved in methanol. Trolox, a well-known antioxidant, is used as the positive control and prepared at the same concentrations for comparison. To conduct the assay, 100 µL of each sample or Trolox solution is mixed with 3 mL of freshly prepared FRAP reagent in a 96-well plate or test tube. The mixtures are incubated at 37 °C for 30 min, allowing the antioxidants in the samples to reduce the Fe^3^⁺ ions to Fe^2^⁺. After incubation, the absorbance of the resulting blue-colored complex is measured at 593 nm using a UV–visible spectrophotometer. The FRAP value of each sample is calculated by comparing the absorbance to a standard curve generated using known concentrations of ferrous sulfate (FeSO₄). The antioxidant capacity is expressed as µmol Fe^2^⁺ equivalents/g of the sample. The experiment included three separate readings for every measurement.

### SARS-CoV-2 antiviral potential evaluation

To examine the antiviral potential of the selected compounds, we used an in vitro pre-post-infection approach, treating cells after and before virus adsorption for 1 h, respectively. It was used to seed Vero CCL-81 cells in 96-well plates and to incubate them for 24 h at 37 °C and 5% CO_2_. After that, the compounds were prepared in dilutions selected based on a cytotoxicity test and added to the monolayer of cells (50 µL /well) for 1 h. After pre-treatment, 50 µL of DMEM supplemented with 2% FBS was added to each well, the compound was removed, and the virus inoculum was added at 0.01 multiplicity of infection. 150 µL /well of the pre-treatment dilutions of the virus were added to the wells after 1 h at 37 °C, and adsorption was completed after 1 h at 37 °C. In the final experiment, A 5% CO_2_ environment was used to incubate the plates for 48 h at 37 °C (Araujo et al. 2020). It was decided to determine the viral titer by plaque assay by harvesting the supernatants after 48 h of incubation and storing them at 80 °C for 48 days. A positive inhibition control, Remdesivir (RDV), was included in our study at 100 µg/mL. A control for non-infected cells was obtained using the supernatant of infected cells without any treatment (Jang et al. 2021).

### Plaque assay

Compounds were assessed for their antiviral potential counter to SARS-CoV-2 by plaque assay. We planted 1.2 × 10^5^ Vero CCL-81 cells per well throughout 24 h and kept the cells at 37 °C and with 5% CO_2_. The supernatants from the antiviral assay were diluted tenfold on a serial basis (200 µL/well) and were then added to a monolayer of cells and incubated for 1 h at 37 °C with 5% CO_2_ before the dilutions were added to the monolayers. Then, 1 mL of semi-solid medium (1.5% carboxymethylcellulose in DMEM 1 × with 2% FBS and 1% penicillin–streptomycin) was added to the wells after removing the viral inoculum. Three days of incubation were carried out at 37 °C, with 5% CO_2_ in the atmosphere. Once the monolayers were washed two times with PBS, they were adhered to and dyed with 4% formaldehyde and 1% crystal violet, and the number of viral plaques was recorded (Straková et al. 2023). The assay was performed in triplicate to ensure reproducibility and statistical analysis.

### Quantification of cytokines in propolis-treated cells supernatants

To investigate the effects of the selected compounds on cytokine production, we focused on propolis due to its observed antiviral activity and potential immunomodulatory properties in the previously infected Vero CCL-81 culture supernatants. Propolis-treated Vero CCL-81 cells were analyzed for TNF-β, IL-1β, and IL-10 concentrations. Ten thousand cells for Vero CCL-81 were planted in 96-well plates. After 24 h of adhesion, the cells were treated with a 9 µg/mL propolis sample for 3 h (Berretta et al. 2020). The cytokine levels in the culture supernatants were measured using the enzyme-linked immunosorbent assay (ELISA). Following the manufacturer's instructions, a unique ELISA kit (bioscience, San Diego, CA, USA) was used for each cytokine. The optical density of the reaction products was evaluated using an ELISA plate reader (EnSpire Multimode Plate Reader).

### In silico assessment

The three-dimensional structures of main protease (6lu7), spike protein S1 (7l4z), and RNA-directed RNA polymerase (7bv2) of SARS-CoV-2 were retrieved from RCSB Protein Data Bank (RCSB PDB; https://www.rcsb.org/) database. In addition, the three-dimensional structures of propolis curcumin, quercetin, and ginseng's active ingredients were obtained from PubChem (https://pubchem.ncbi.nlm.nih.gov/) database. Protein structures' preparation and docking processes were finalized using Molecular Operating Environment (MOE 2022.02, Chemical Computing Group, Montreal, QC, Canada) software. The docking procedure followed established protocols described in previous studies (Kraiem et al. 2024). Furthermore, The docking procedure followed established protocols, employing the following steps: The protein structures were prepared by removing water molecules and adding missing hydrogen atoms. The ligand structures were prepared by removing salts and adding missing hydrogen atoms. The prepared ligands were docked into the binding sites of the target proteins using the Triangle Match algorithm. The docked poses were scored using the London dG scoring function, which calculates the binding energy based on van der Waals interactions, hydrogen bonding, electrostatic interactions, and desolvation energy.

### Analyses of statistics

GraphPad Prism version 9.0.5 (La Jolla, CA, USA) was used to analyze all the data. We presented our data as mean ± SEM. The IC_50_ values were calculated using a four-parameter logistic regression model, commonly used for fitting dose–response curves. A t-student test and one-way ANOVA were followed by Tukey's post-hoc test to compare the effects of different compound concentrations on the control groups. As a rule, *p*-values of 0.05 or less are considered significant.

## Results

### Cytotoxicity assay to figure out the non-cytotoxic dose for all compounds

**Fig. 1:**
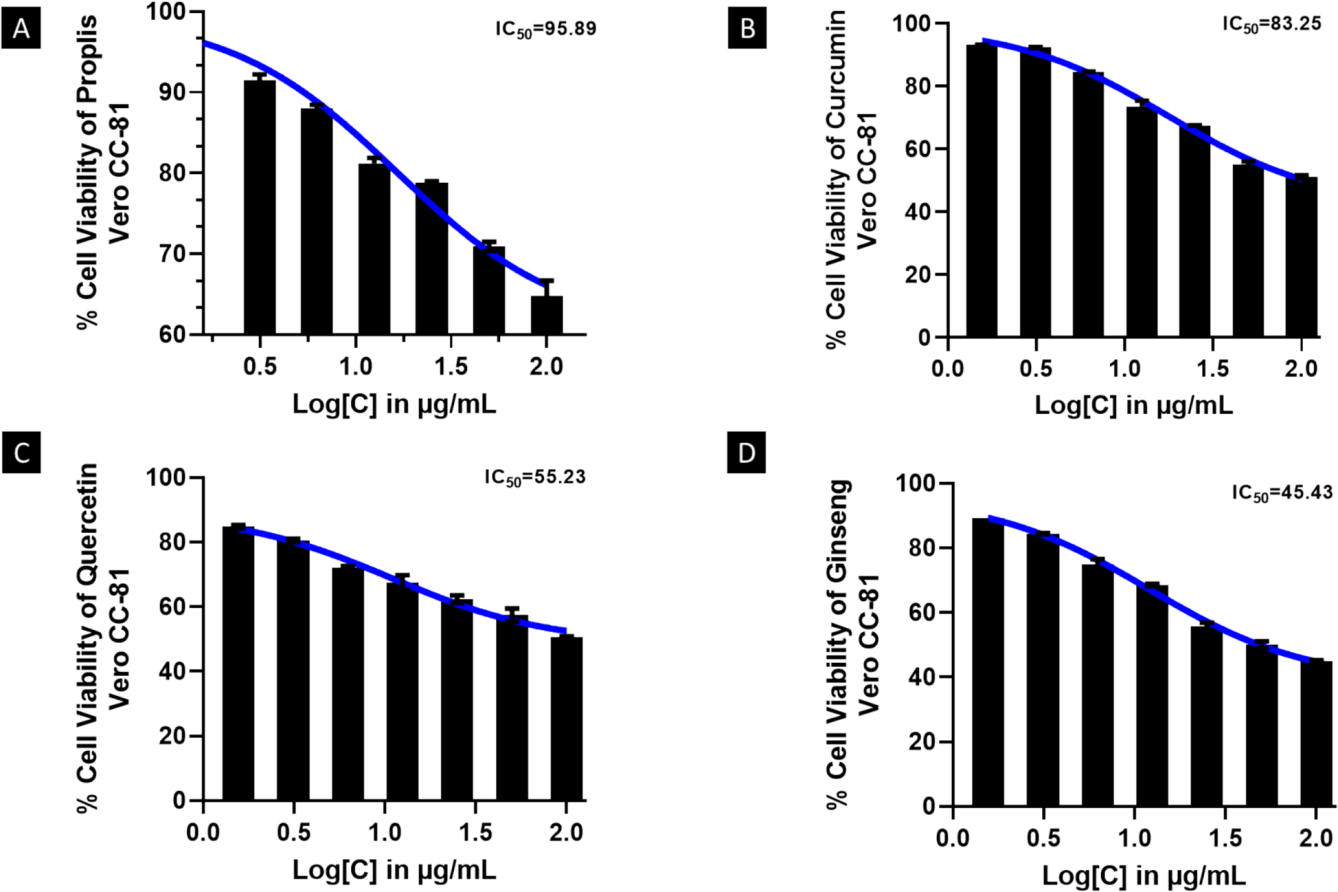
Tests on Vero CCL-81 cells for cytotoxicity of the selected compounds. After 48 h of treatment with the chosen compounds at five different concentrations, Vero CCL-81 monolayers were analyzed for viability. Statistical data is presented as a mean ± SEM. The graphs revealed that the selected compounds have a dose-dependent cytotoxic effect on Vero CCL-81 cells, with the highest concentration of each compound resulting in mild low cell viability. This indicated that these compounds could be potentially used as anti-COVID-19 agents. All experiments were repeated at least three times

A concentration range of 100, 50, 25, 12.5, 6.25, 3.125, and 1.56 µg/mL were tested to identify the compounds' cytotoxicity dose. Neither 6.25 nor 12.5 µg/mL of the compound asserted injury to the monolayer; however, higher concentrations induced morphological alterations, notably rounding and loss of adhesion. This data indicated that the compounds have a threshold concentration below which they are not toxic, and higher concentrations can cause cellular damage. To test the antiviral potential of the compounds versus SARS-CoV-2, concentrations of 6.25 and 12.5 µg/mL were selected. Additionally, MTT assays were performed on all compounds to assess cell viability. It was observed that the viability of the cells was 98.31, 92.32, 88.22, 81.23, 78.62, 70.24, and 66.20% at propolis concentrations of 1.56, 3.125, 6.25, 12.50, 25, 50, and 100 µg/mL, with an IC_50_ of 95.89 µg/mL, as revealed in (Fig. 1a). The results suggested that propolis is an effective antiviral agent against SARS-CoV-2, with an IC_50_ of 95.89 µg/mL. Therefore, it can be considered a safe and effective therapeutic agent for COVID-19. Furthermore, the three consecutive effective drugs that have good viability in this context are curcumin, quercetin, and ginseng, which were found to have IC_50_ values of 83.15, 55.23, and 45.43 µg/mL, respectively, as outlined in (Fig. 1b–d).

### Analysis of fraction inhibition

**Fig. 2:**
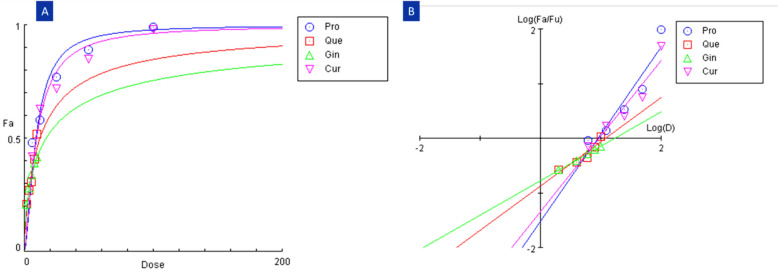
A dose–effect plot and a median-effect plot are revealed in the *CompuSyn* graphs for the selected compounds: **A** Dose–effect plot; **B** Median-effect plot. The dose–effect plot revealed that the combined effects of propolis and curcumin are greater than those of either compound alone. The median-effect plot also revealed a non-linear relationship between the two compounds, indicating that the two compounds have a strong synergistic effect. "Pro," "Que," "Gin," and "Cur" are abbreviations for propolis, quercetin, ginseng, and curcumin, respectively

As can be seen in (Fig. 2a, b), *CompuSyn* blots revealed the results of the analysis. It was noted that different doses of the compounds resulted in significantly different viability levels in Vero CCL-81 cells. Based on the dosage effects of the compounds administered to varying dosages for 48 h, 0.5 fraction inhibition (Fa) was observed, and dose effect values were tabularized in (Table 2), which evaluated dosage reduction and the impact of the compound. The graph revealed that the compounds had varying effects on cell viability, with the highest concentration of the compound resulting in the highest Fa. Table 2 also exhibited that as the dose of the compound decreased, the Fa decreased, indicating that the compounds had dose-dependent effects on cell viability.

**Table 2:** Dose-dependent of the different compounds on Vero cell growth after 48 h treatment at the different fraction affected

	Fraction affected (Fa)	Dose			
		Propolis	Curcumin	Quercetin	Gensing
Compound	0.5	9.06407	9.42125	12.0260	16.7892
	0.75	17.9794	20.7718	46.3852	95.2555
	0.9	35.6637	45.7973	178.911	540.444
	0.95	56.8246	78.4112	448.103	1759.89
	0.97	79.1561	114.960	861.173	4076.67

Analyses were conducted using *CompuSyn* software using dose and effect data from the MTT assay. *CompuSyn* software is a powerful tool for analyzing the dose–response relationship of a drug, as well as its ability to cause an effect on cells

### Investigating the antioxidant capacity: a triad approach with ABTS, CUPRAC, and FRAP assays

The antioxidant capacities of propolis, curcumin, quercetin, and ginseng were evaluated using the FRAP, CUPRAC, and ABTS assays at concentrations of 10, 20, 40, 80, and 100 µg/mL, as revealed in Table 3. Propolis exhibited the highest antioxidant activity across all assays, with a maximum FRAP value of 1200.55 ± 15.90 μmol Fe^2^⁺ eq/g, a CUPRAC value of 1150.80 ± 14.20 μmol Trolox eq/g, and an ABTS value of 1250.40 ± 17.10 μmol Trolox eq/g at the 100 µg/mL concentration. Curcumin displayed moderate antioxidant activity, achieving a maximum FRAP value of 650.25 ± 11.20 μmol Fe^2^⁺ eq/g, a CUPRAC value of 630.80 ± 10.50 μmol Trolox eq/g, and an ABTS value of 670.20 ± 12.70 μmol Trolox eq/g at the same concentration. Quercetin also demonstrated significant antioxidant effects, with maximum values of 800.45 ± 12.50 μmol Fe^2^⁺ eq/g in FRAP, 780.55 ± 11.20 μmol Trolox eq/g in CUPRAC, and 820.30 ± 14.00 μmol Trolox eq/g in ABTS, while ginseng showed the least antioxidant capacity among the samples tested, with values of 700.60 ± 12.30 μmol Fe^2^⁺ eq/g in FRAP, 680.50 ± 11.20 μmol Trolox eq/g in CUPRAC, and 720.30 ± 13.50 μmol Trolox eq/g in ABTS. The significant antioxidant activity of propolis, along with its consistently high values in all assays, underscores its potential as a natural source of antioxidants.

**Table 3:** FRAP, CUPRAC, ABTS antioxidant assay results of propolis, curcumin, quercetin, and ginseng

Samples	Concentration (µg/mL)	FRAP (μmol Fe^2^⁺ eq/g) ± SD	CUPRAC (μmol Trolox eq/g) ± SD	ABTS (μmol Trolox eq/g) ± SD	P-value (FRAP vs Trolox)	P-value (CUPRAC vs Trolox)	P-value (ABTS vs Trolox)
Propolis	10	200.45 ± 5.00	195.30 ± 4.80	210.50 ± 5.30	0.01	0.02	0.01
	20	350.15 ± 7.00	340.10 ± 6.80	370.20 ± 8.00	0.005	0.01	0.005
	40	600.30 ± 10.50	580.20 ± 9.70	620.10 ± 11.20	< 0.001	0.005	0.001
	80	900.10 ± 12.80	860.50 ± 11.00	900.30 ± 13.50	< 0.001	< 0.001	< 0.001
	100	1200.55 ± 15.90	1150.80 ± 14.20	1250.40 ± 17.10	< 0.001	< 0.001	< 0.001
Curcumin	10	100.20 ± 3.80	95.25 ± 3.50	110.30 ± 3.90	0.05	0.06	0.04
	20	180.20 ± 4.90	175.30 ± 5.10	200.10 ± 5.60	0.03	0.04	0.02
	40	300.15 ± 6.50	290.00 ± 6.00	320.20 ± 7.20	0.02	0.025	0.01
	80	500.45 ± 9.00	490.10 ± 8.70	520.50 ± 10.50	0.01	0.01	0.005
	100	650.25 ± 11.20	630.80 ± 10.50	670.20 ± 12.70	0.005	0.005	0.001
Quercetin	10	120.30 ± 4.00	115.25 ± 3.70	130.40 ± 4.50	0.05	0.06	0.04
	20	200.15 ± 5.20	190.20 ± 5.00	210.10 ± 6.00	0.03	0.04	0.02
	40	350.45 ± 7.50	340.30 ± 7.00	360.40 ± 8.00	0.02	0.02	0.01
	80	600.55 ± 10.10	590.10 ± 9.50	620.20 ± 11.50	0.005	0.005	0.002
	100	800.45 ± 12.50	780.55 ± 11.20	820.30 ± 14.00	0.002	0.003	0.001
Ginseng	10	90.25 ± 3.40	85.50 ± 3.20	100.30 ± 3.70	0.06	0.07	0.05
	20	150.80 ± 4.50	140.20 ± 4.00	160.10 ± 4.80	0.04	0.05	0.03
	40	300.20 ± 5.80	290.25 ± 5.50	310.10 ± 6.40	0.03	0.04	0.02
	80	500.50 ± 9.10	490.30 ± 8.60	520.40 ± 10.20	0.01	0.01	0.005
	100	700.60 ± 12.30	680.50 ± 11.20	720.30 ± 13.50	0.005	0.005	0.001
Trolox (positive control, 1000 µg/mL)	–	1200.00 ± 20.00	1100.00 ± 17.50	1300.00 ± 22.00	–	–	–

### The antiviral potential of the selected compounds against SARS-CoV-2

**Fig. 3:**
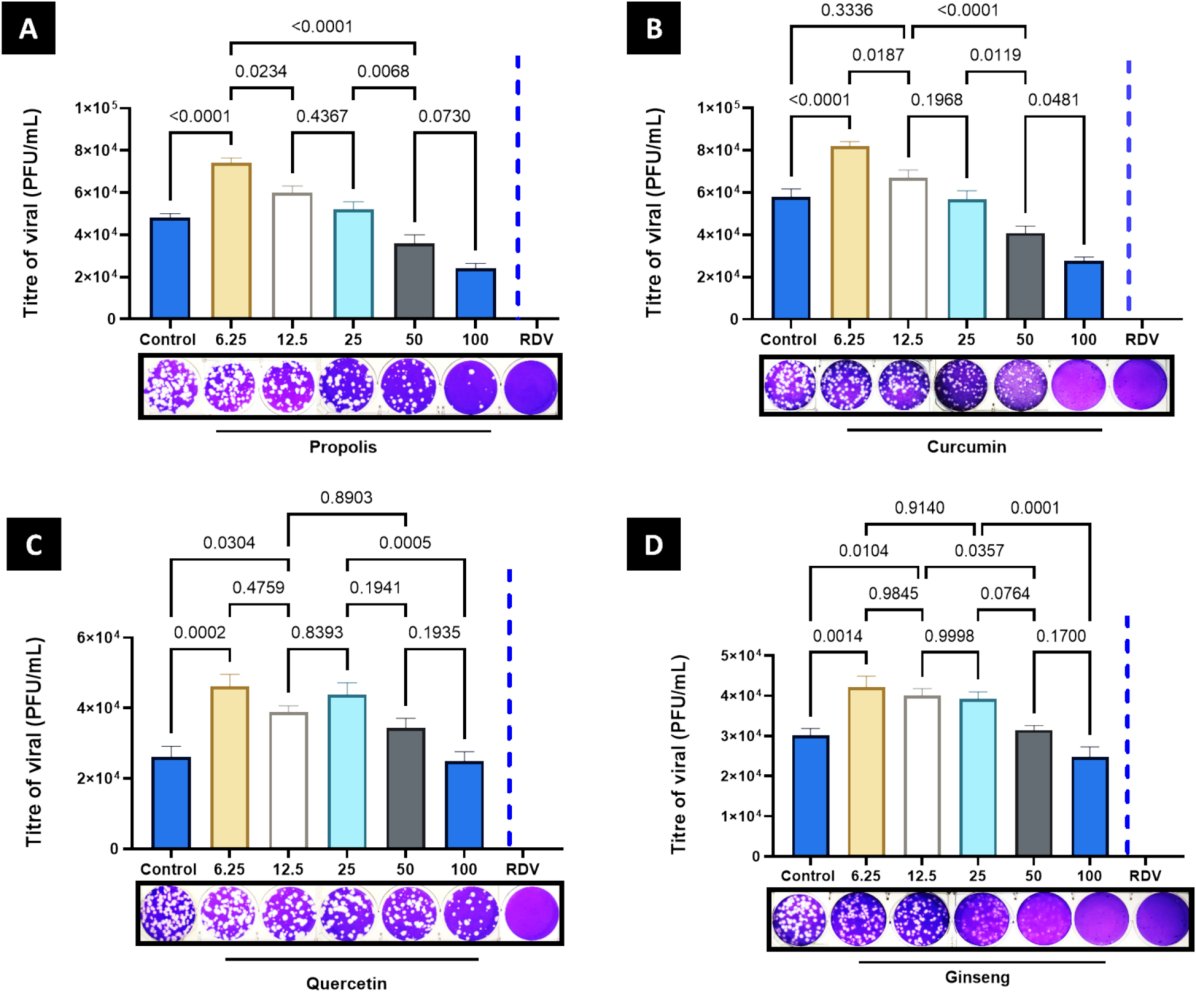
SARS-CoV-2 antiviral activity of different compounds in Vero CCL-81. SARS-CoV-2 levels in supernatants of Vero CCL-81 monolayers were compared with titers in plaque assays done after 5 days of incubation with the selected compounds. A bar represents a mean level of data (± SEM). The significance of the differences was determined using a one-way analysis of variance followed by Tukey's multiple comparison test. Each experiment consisted of three replicas. *RDV* Remdesivir

After 48 h of pre-post treatment strategy, compounds were found to significantly reduce the amount of SARS-CoV-2 virus in the Vero CCL-81 supernatants compared to the untreated control. Propolis compounds exhibited a notable decrease in viral titer at concentrations of 100 and 50 µg/mL, according to (Fig. 3a). Similarly, treatment with curcumin also reduced viral titer at 100 and 50 µg/mL (Fig. 3b). This suggested that the compounds could interfere with the viral replication cycle by directly inhibiting the virus or stimulating the immune system. Both compounds effectively reduced the virus at concentrations as low as 50 µg/mL, indicating they are likely effective for treating SARS-CoV-2 infections. However, the antiviral properties of quercetin and ginseng were limited to only the 100 µg/mL concentration. There was no noticeable change in viral titer after treating quercetin and ginseng for 48 h at 6.25, 12.5, 25, and 50 µg/mL concentrations (Fig. 3c, d). This data suggested that quercetin and ginseng are effective antiviral agents only at high concentrations. This could be due to the low solubility of these compounds, which prevents them from being effectively absorbed into cells at lower concentrations. RVD at 100 µg/mL was an effective inhibition control, screening a 100% decrease in SARS-CoV-2 viral titer, as depicted in (Fig. 3). This revealed that RVD is highly soluble and can easily penetrate cells, making it an effective antiviral agent at lower concentrations than the other compounds.

### Cytokines released by propolis-treated cells

**Fig. 4:**
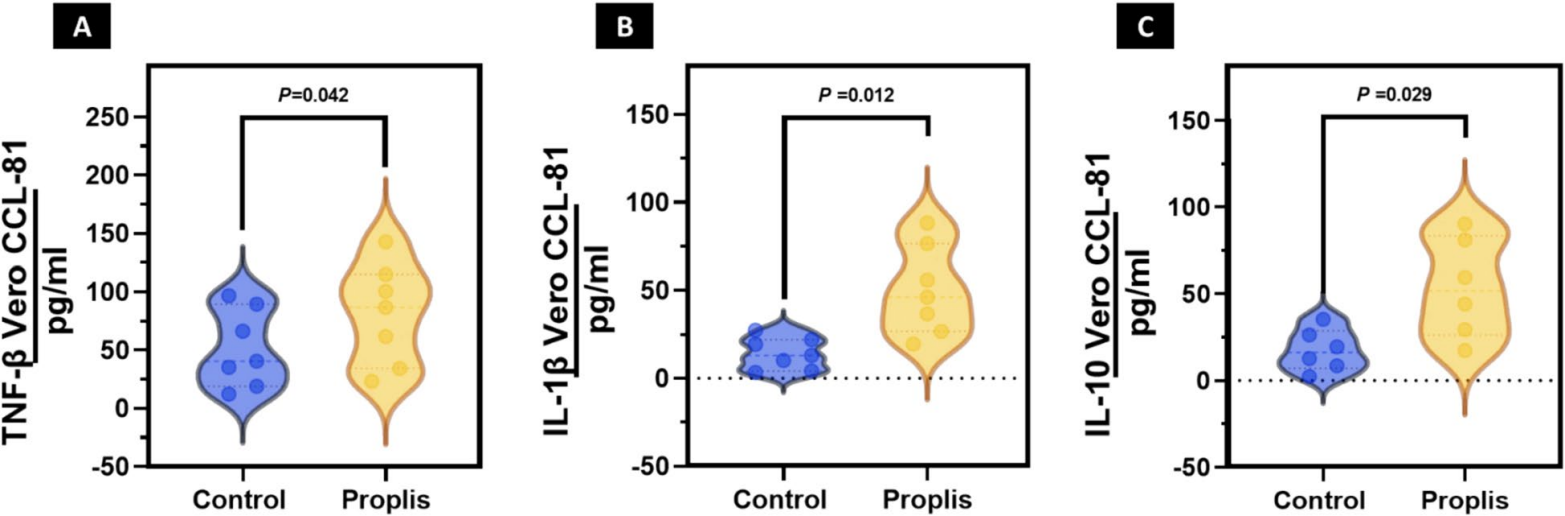
Propolis treatment induces the production of pro-inflammatory cytokines by Vero CCL-81 cells. **A** Production of TNF-β. **B** Vero CCL-81 cells were treated with IL-1β and propolis. **C** IL-10 and Propolis-treated Vero CCL-81 cells. Cells were treated with 25 μg/mL propolis for 2 h. In culture supernatants, cytokine levels were measured by ELISA. The results are presented as the mean SEM of two independent experiments (*p* < 0.05, Student's t-test)

Interestingly, propolis was the most prominent compound that resulted in a high reduction in the viral title compared to the other compounds. Therefore, a more in-depth investigation is being performed to assess whether propolis can reduce the cytokine storm that results from the infection of COVID-19 after the post-infection period. From our data, the propolis stimulates the secretion of TNF-β, IL-1β, and IL-10 in Vero CCL-81 cells, as revealed in (Fig. 4a–c).

### Insilico studies assessment

**Fig. 5:**
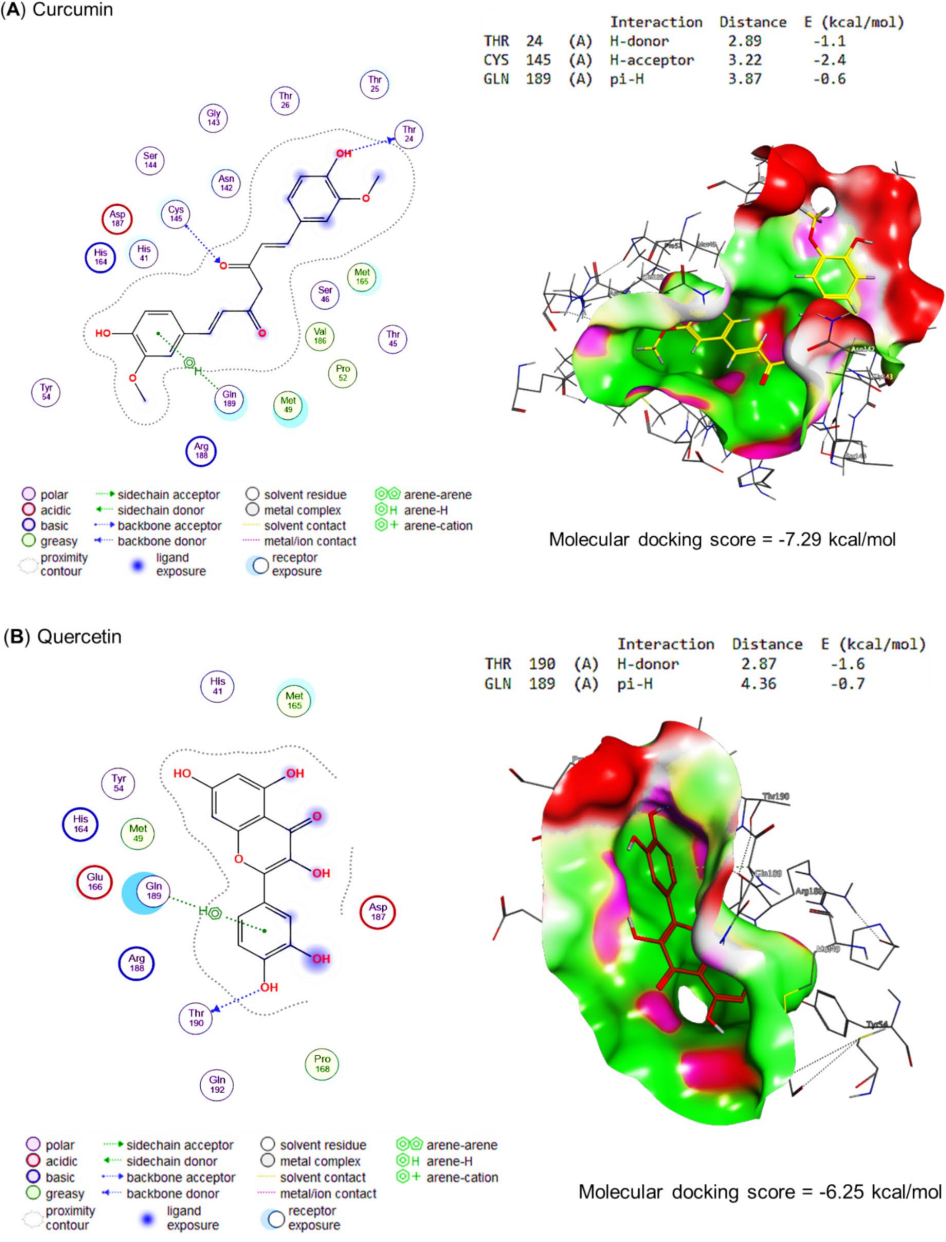

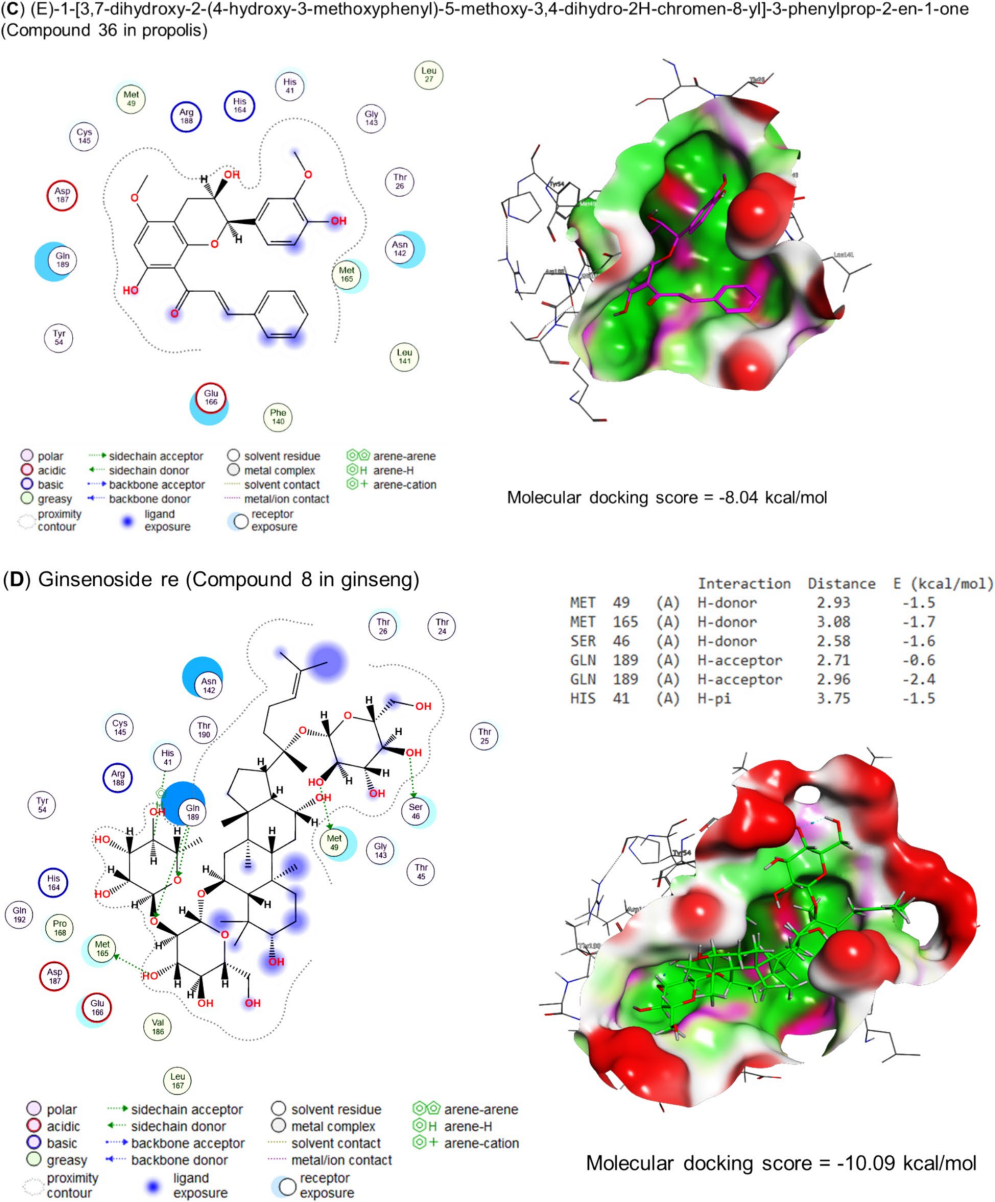
Molecular docking scores as main protease inhibitors for **A** curcumin, **B** quercetin, **C** propolis, and **D** ginseng's bioactive compounds. The figure revealed that the molecular docking scores for curcumin, quercetin, propolis, and ginseng were −7.29, −6.25, −8.04, and −10.09 kcal/mol, respectively. These scores demonstrate the potential of these bioactive compounds as main protease inhibitors

**Fig. 6:**
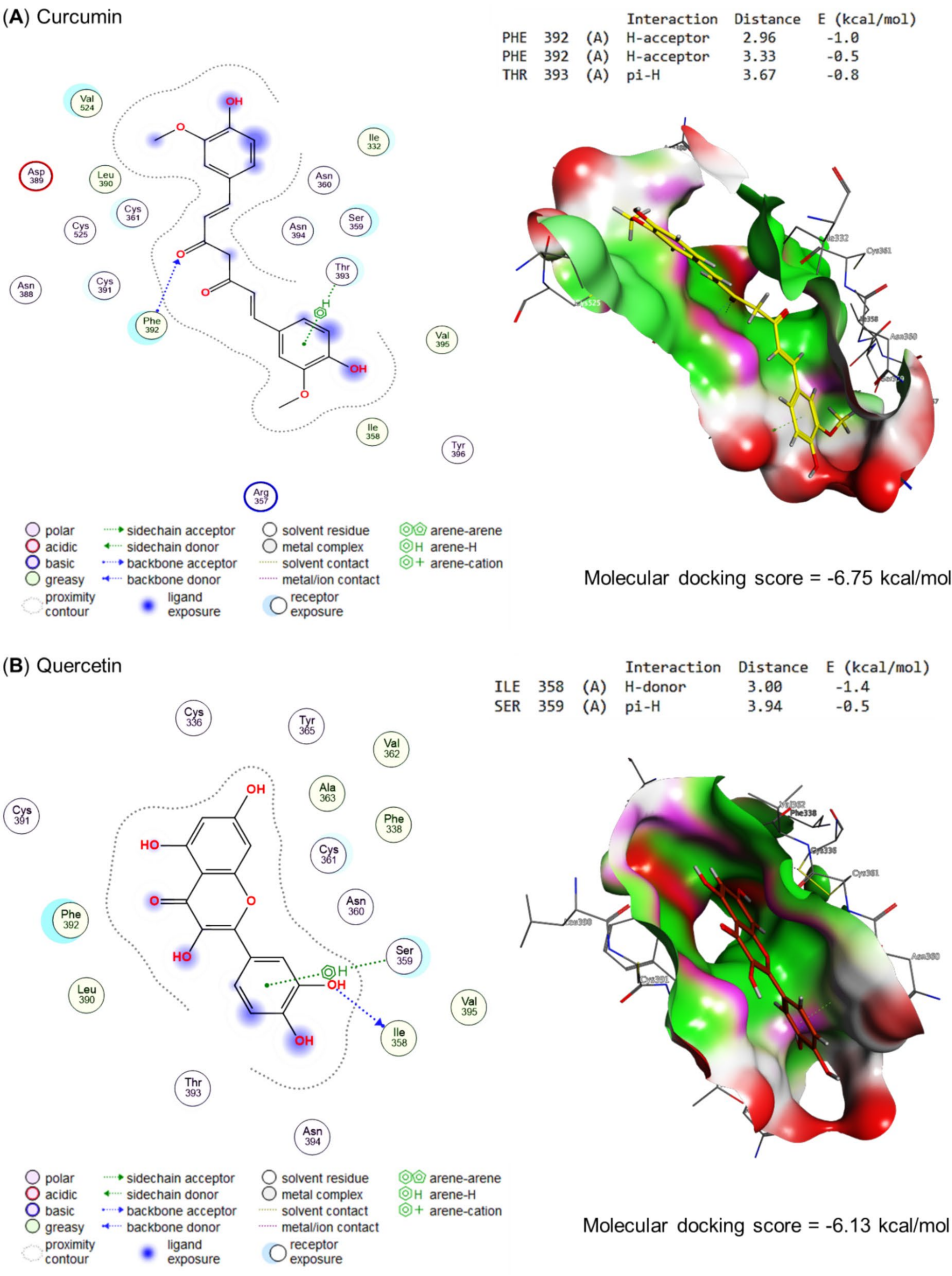

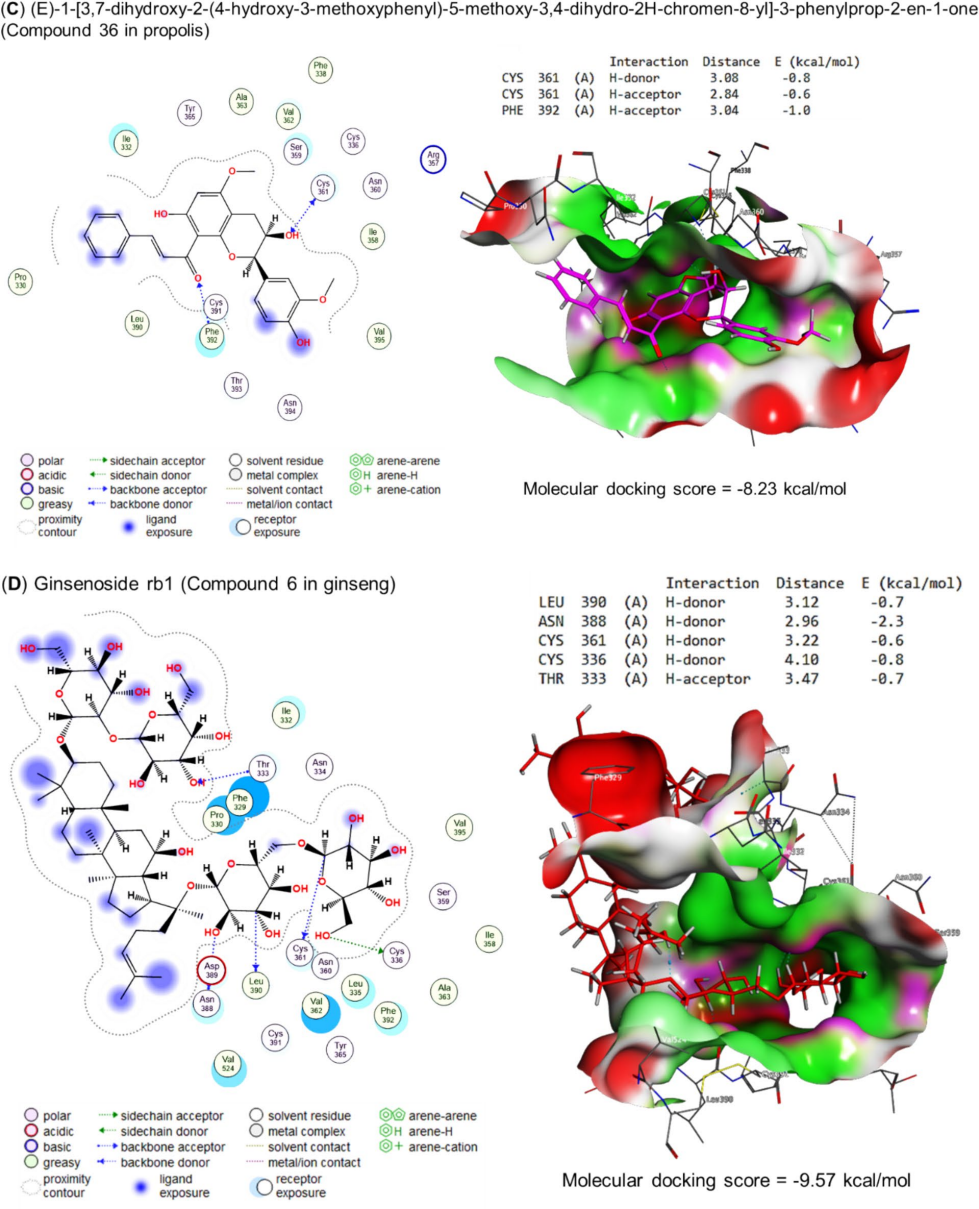
Molecular docking scores as Spike protein S1 inhibitors for **A** Curcumin, **B** Quercetin, **C** Propolis, and **D** Ginseng's bioactive compounds The figure revealed that the molecular docking scores for curcumin, quercetin, propolis, and ginseng were −6.75, −6.13, −8.23, and −9.57 kcal/mol, respectively. These scores represent the energy needed to bind the molecules to the spike protein S1. A negative score indicates that less energy is needed to bind the molecule, which is beneficial for inhibiting the spike protein

**Fig. 7:**
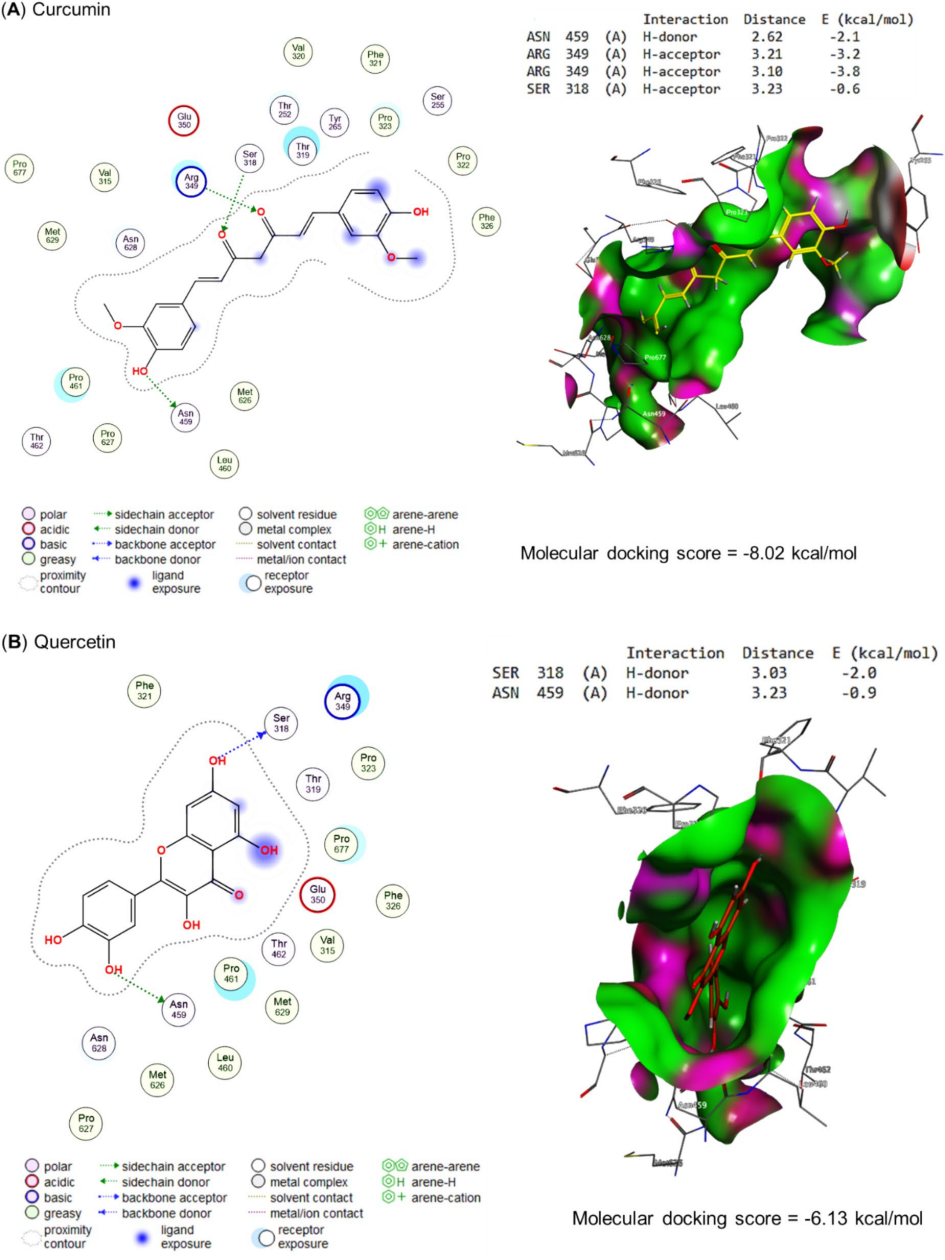

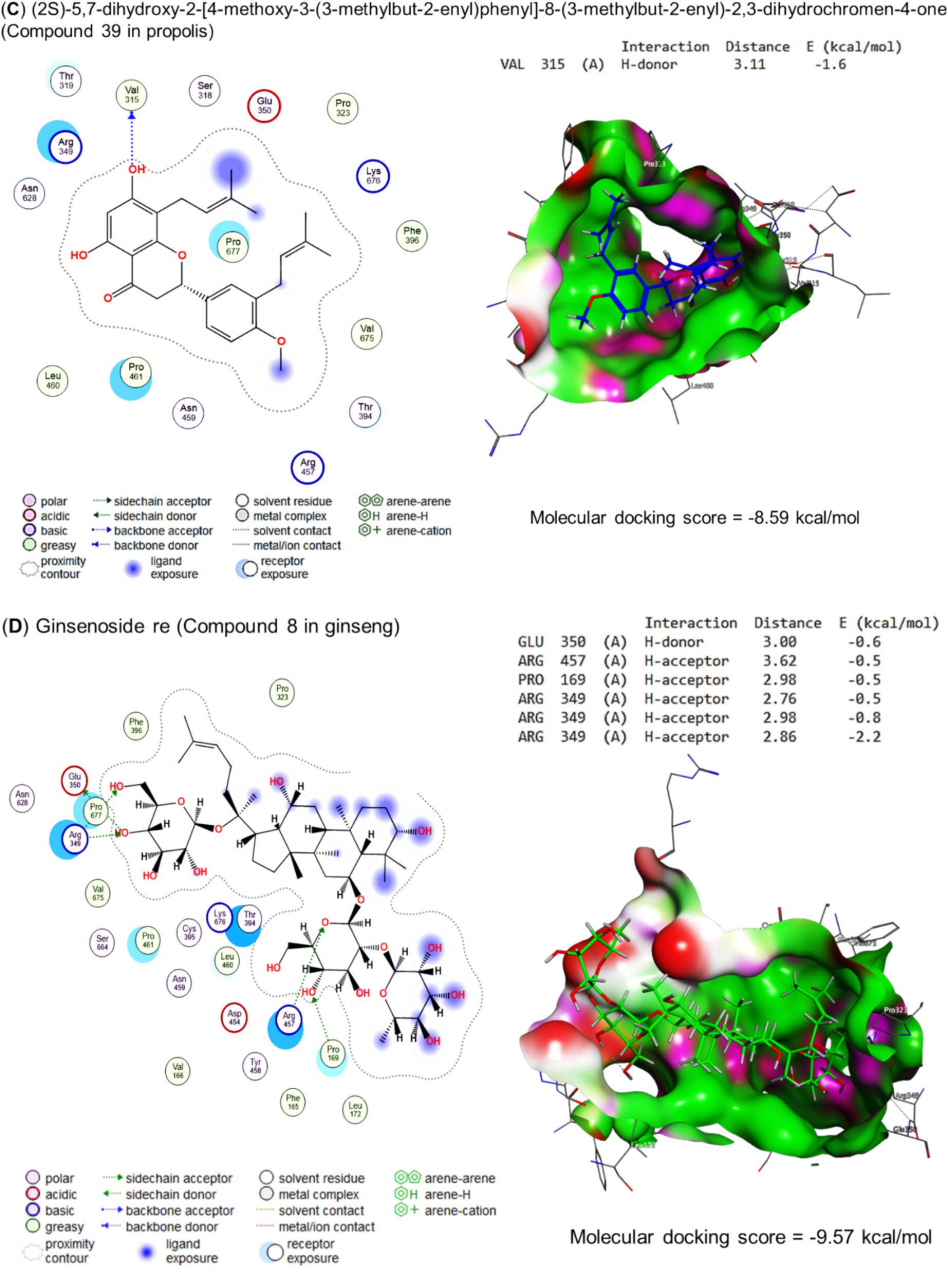
Molecular docking scores as RNA polymerase inhibitors for **A** Curcumin, **B** Quercetin, **C** Propolis, and **D** Ginseng's bioactive compounds. The figure revealed that the molecular docking scores for curcumin, quercetin, propolis, and ginseng were −8.02, −6.13, −8.59, and −9.57 kcal/mol, respectively. These scores show the potential of these compounds to inhibit the RNA polymerase, an enzyme required for the transcription of genetic information. The scores indicate that these compounds may be able to bind to the RNA polymerase and disrupt its activity

Molecular docking of propolis, curcumin, quercetin, and the bioactive compounds of ginseng against main protease, spike protein S1, and RNA-directed RNA polymerase of SARS-CoV-2 are represented in Table 4 and Figs. 5, 6, and 7, respectively. Curcumin interacted with THR24 (H-donor), CYS145 (H-acceptor), and GLN189 (pi-H) residues in the binding site of the main protease with an energy of -7.29 kcal/mol (Fig. 5A). Also, quercetin interacted with THR190 (H-donor) and GLN189 (pi-H) residues in the binding site of the main protease by an energy value of −6.25 kcal/mol (Figs. 5B). (E)-1-[3,7-dihydroxy-2-(4-hydroxy-3-methoxyphenyl)-5-methoxy-3,4-dihydro-2H-chromen-8-yl]-3-phenylprop-2-en-1-one, the bioactive compound of propolis effectively bound with the binding site of main protease by energy value of -8.04 kcal/mol (Fig. 5C). Ginsenoside re, the bioactive compound present in ginseng, expressed a higher energy (−10.09 kcal/mol) for interaction with MET48 (H-donor), MET165 (H-donor), SER46 (H-donor), GLN (two H-acceptors), and HIS41 (pi-H) residues in the binding site of main protease (Fig. 5C).

**Table 4:** Molecular docking scores of propolis, curcumin, quercetin, and ginseng’s bioactive compounds

Compounds	NO	PubChem CID	Molecular docking scores		
			Main protease	Spike protein S1	RNA-directed RNA polymerase
Curcumin		969516	−7.29	−6.75	−8.02
Quercetin		5280343	−6.25	−6.13	−6.85
Propolis					
7-Hydroxyflavanone	1	1890	−6.00	−5.89	−6.27
Pinocembrin	2	68071	−6.16	−5.73	−6.54
Pinostrobin	3	73201	−6.28	−5.96	−6.19
Pinobanksin	4	73202	−6.20	−5.90	−6.04
Vestitol	5	92503	−6.20	−6.25	−7.05
Euchrestaflavanone A	6	484588	−7.82	−7.81	−7.42
Sophoraflavanone B	7	509245	−6.44	−6.71	−6.98
5-Methoxy-3,7-dihydroxyflavanone	8	597405	−6.31	−6.03	−6.29
Propolin C	9	639465	−7.45	−7.97	−8.20
5-Methoxy-7-hydroxyflavanone	10	4053302	−6.47	−6.23	−6.69
4-Hydroxy-3,9-dimethoxypterocarpan	11	4484393	−6.78	−6.01	−7.19
Chrysin	12	5281607	−5.96	−5.58	−6.00
Kaempferide	13	5281666	−6.22	−6.11	−6.46
Rhamnetin	14	5281691	−6.55	−6.25	−6.65
Caffeic acid phenethyl ester	15	5281787	−6.49	−6.09	−6.66
Phenethyl Ferulate	16	5284444	−6.57	−6.54	−6.45
Melilotocarpan D	17	5319351	−6.53	−6.79	−6.57
7,5',6'-trihydroxy-4'-methoxyisoflavan	18	5326316	−6.55	−6.46	−7.43
Galangin-5-methylether	19	5488105	−6.20	−5.96	−6.34
Benzyl caffeate	20	5919576	−5.83	−6.46	−6.67
Benzyl ferulate	21	7766,335	−6.40	−6.75	−6.67
Culifolin	22	9861070	−7.18	−6.58	−8.00
(Z)-3-[2,2-Dimethyl-8-(3-methyl-2-butenyl)-2H-1-benzopyran-6-yl]propenoic acid	23	9948201	−6.99	−6.24	−7.59
Isopentyl caffeate	24	10264116	−6.27	−6.07	−6.60
Nymphaeol C	25	10323393	−7.79	−7.72	−7.58
Nymphaeol B	26	10387631	−7.99	−7.40	−7.37
Isomangiferolic acid	27	14034468	−6.95	−7.35	−8.22
Mangiferonic acid	28	14034474	−7.11	−7.88	−7.43
3beta-Hydroxycycloart-24-en-27-al	29	14314541	−6.89	−7.11	−6.93
(E,6S)-5-hydroxy-6-[(1S,3R,6R,8R,11S,12S,15R,16R)-6-hydroxy-7,7,12,16-tetramethyl-15-pentacyclo[9.7.0.01,3.03,8.012,16]octadecanyl]-2-methylhept-2-enoic acid	30	14314559	−7.20	−7.82	−7.83
(E,6R)-4-hydroxy-6-[(1S,3R,6S,8R,11S,12S,15R,16R)-6-hydroxy-7,7,12,16-tetramethyl-15-pentacyclo[9.7.0.01,3.03,8.012,16]octadecanyl]-2-methylhept-2-enoic acid	31	14314562	−6.82	−7.48	−7.86
(24E)-3alpha,27-Dihydroxycycloarta-24-ene-26-oic acid	32	14314,564	−6.94	−7.12	−7.81
Isopentyl ferulate	33	14589,006	−6.25	−6.19	−6.25
(2R,3S)-2-(3,5-Dihydroxyphenyl)-5-methoxy-8-[(E)-3-phenylpropenoyl]-3,4-dihydro-2H-1-benzopyran-3,7-diol	34	42638982	−7.78	−7.38	−7.18
(2S,3R)-2-(3,5-Dihydroxyphenyl)-5-methoxy-8-[(E)-3-phenylpropenoyl]-3,4-dihydro-2H-1-benzopyran-3,7-diol	35	42638983	−7.86	−7.40	−7.49
(E)-1-[3,7-dihydroxy-2-(4-hydroxy-3-methoxyphenyl)-5-methoxy-3,4-dihydro-2H-chromen-8-yl]-3-phenylprop-2-en-1-one	36	44188970	−8.04	−8.23	-−7.75
(24E)-3-Oxo-27,28-dihydroxycycloarta-24-ene-26-oic acid	37	44234436	−7.44	−7.48	−7.35
(2Z,4E,6R)-2-methyl-6-[(1S,3R,8R,11S,12S,15R,16R)-7,7,12,16-tetramethyl-6-oxo-15-pentacyclo[9.7.0.01,3.03,8.012,16]octadecanyl]hepta-2,4-dienoic acid	38	45268374	−6.92	−7.16	−6.94
(2S)-5,7-dihydroxy-2-[4-methoxy-3-(3-methylbut-2-enyl)phenyl]-8-(3-methylbut-2-enyl)-2,3-dihydrochromen-4-one	39	45268397	−7.72	−7.33	−8.59
27-Hydroxymangiferolic acid	40	45269256	−7.27	−7.05	−6.78
Mangiferolic acid	41	45270099	−7.17	−7.96	−7.12
28-hydroxymangiferonic acid	42	45270931	−7.15	−6.79	−7.93
27-hydroxymangiferonic acid	43	45270932	−7.70	−7.30	−7.79
23-Hydroxymangiferonic acid	44	45271778	−7.05	−7.53	−7.67
(S)-5,7-dihydroxy-2-(4-methoxyphenyl)-8-(3-methylbut-2-enyl)chroman-4-one	45	45272659	−7.45	−6.92	−6.88
2,2-dimethyl-6-carboxyethenyl-8-prenyl-2 h-1-benzopyran	46	54552371	−7.49	−6.50	−7.16
(2S)-2-[2-(3,7-dimethylocta-2,6-dienyl)-3,4-dihydroxyphenyl]-5,7-dihydroxy-6-(3-methylbut-2-enyl)-2,3-dihydrochromen-4-one	47	91152580	−7.66	−7.59	−8.33
(2S)-2-[2-(3,7-dimethylocta-2,6-dienyl)-3,4-dihydroxyphenyl]-5,7-dihydroxy-2,3-dihydrochromen-4-one	48	91159164	−7.93	−7.49	−7.23
(2S)-2-(3,4-dihydroxyphenyl)-6-(3,7-dimethylocta-2,6-dienyl)-5,7-dihydroxy-2,3-dihydrochromen-4-one	49	91461568	−7.18	−7.54	−7.65
(6R)-2-(hydroxymethyl)-6-[(1S,3R,6R,8R,11S,12S,15R,16R)-6-hydroxy-7,7,12,16-tetramethyl-15-pentacyclo[9.7.0.01,3.03,8.012,16]octadecanyl]hept-2-enoic acid	50	162895344	−6.57	−7.01	−7.38
(6R)-2-(hydroxymethyl)-6-[(1S,3R,6S,8R,11S,12S,15R,16R)-6-hydroxy-7,7,12,16-tetramethyl-15-pentacyclo[9.7.0.01,3.03,8.012,16]octadecanyl]hept-2-enoic acid	51	162895345	−7.27	−7.43	−7.45
(6R)-2-(hydroxymethyl)-6-[(1S,3R,8R,11S,12S,15R,16R)-7,7,12,16-tetramethyl-6-oxo-15-pentacyclo[9.7.0.01,3.03,8.012,16]octadecanyl]hept-2-enoic acid	52	162920419	−6.78	−7.02	−7.53
(6R)-2-(hydroxymethyl)-6-[(1S,3R,7R,8R,11S,12S,15R,16R)-7-(hydroxymethyl)-7,12,16-trimethyl-6-oxo-15-pentacyclo[9.7.0.01,3.03,8.012,16]octadecanyl]hept-2-enoic acid	53	162971214	−7.45	−7.28	−7.27
(3R)-3,7-dihydroxy-5-methoxy-2-phenyl-2,3-dihydrochromen-4-one	54	162977970	−6.43	−6.01	−6.65
(3R)-3,5-dihydroxy-7-methoxy-2-phenyl-2,3-dihydrochromen-4-one	55	163017232	−6.29	−6.02	−7.22
Ginseng					
α-cedrene	1	6431015	−4.94	−4.99	−4.87
Andrographolide	2	5318517	−6.80	−6.03	−7.03
Cuparene	3	86895	−5.78	−4.95	−5.10
Falcarinol	4	5281149	−7.06	−6.86	−7.05
Ginsenoside compound K	5	9852086	−8.13	−7.75	−8.76
Ginsenoside rb1	6	9898279	−9.72	−9.57	−8.98
Ginsenoside rd	7	11679800	−9.94	−9.30	−9.41
Ginsenoside re	8	3086007	−10.09	−8.63	−9.84
Ginsenoside rg1	9	441923	−8.53	−8.05	−9.76
Ginsenoside rg2	10	21599924	−9.30	−9.07	−9.41
Ginsenoside Rg3	11	9918693	−8.58	−8.58	−9.22
Ginsenoside rh1	12	12855920	−7.37	−8.57	−8.16
Ginsenoside Rh2	13	119307	−7.55	−7.75	−8.91
β-caryophyllene	14	5281515	−4.95	−5.50	−4.95
β-cubebene	15	93081	−5.37	−5.78	−5.84

By the energy of −6.75 kcal/mol, curcumin bound to the PHE392 (two H-acceptors) and THY393 (pi-H) residues in the binding site of spike protein S1, as represented in (Fig. 6A). Also, quercetin interacted by an energy value of −6.13 kcal/mol with ILE358 (H-donor) and SER359 (pi-H) (Fig. 6B). In addition, (E)-1-[3,7-dihydroxy-2-(4-hydroxy-3-methoxyphenyl)-5-methoxy-3,4-dihydro-2H-chromen-8-yl]-3-phenylprop-2-en-1-one, the bioactive compound of propolis interacted with CYS361 (H-donor and H-acceptor) and PH392 (H-acceptor) residues in the binding site of spike protein S1 by energy of −8.23 kcal/mol (Fig. 6C). Furthermore, by H-donor (LEU390, ASN388, CYS361, and CYS336) and H-acceptor (THR333), the bioactive compound of ginseng (ginsenoside rb1) interacted with the binding site of spike protein S1 using energy of −9.57 kcal/mol (Fig. 6D).

Curcumin interacted with RNA-directed RNA polymerase's binding site by H-donor (ASN459 residue), H-acceptor (2, ARG349 and SER318 residues) by energy of −8.02 kcal/mol, as represented in Fig. 7A. In the same binding site, quercetin bound to SER318 and ASN459 by H-donor with an energy of −6.13 kcal/mol (Fig. 7B). With more energy (−8.59 kcal/mol) (2S)-5,7-dihydroxy-2-[4-methoxy-3-(3-methylbut-2-enyl) phenyl]-8-(3-methylbut-2-enyl)-2,3-dihydrochromen-4-one, the bioactive compound of propolis interacted with VAL315 residue by H-donor (Fig. 7C). Moreover, the ginsenoside re (ginseng's bioactive compound) bound to the GLU350, ARG457 (H-acceptor), PRO169 (H-acceptor), and ARG349 (three H-acceptors) residues in the binding site of RNA-directed RNA polymerase with −9.57 kcal/mol, as binding energy (Fig. 7D).

### In silico molecular docking predictions of compound-protein interactions

The in silico molecular docking analysis revealed that the selected compounds exhibited varying degrees of affinity for the main protease, spike protein S1, and RNA-directed RNA polymerase of SARS-CoV-2, as revealed in (Table 5). Among the compounds, ginseng demonstrated the strongest binding affinity for all three proteins, suggesting its potential to interact effectively with these viral targets. Propolis also exhibited strong binding affinities, particularly for the main protease and spike protein S1. Curcumin and quercetin showed moderate binding affinities for the main protease and spike protein S1 but had limited interactions with RNA-directed RNA polymerase. The key residues involved in the interactions between the compounds and the viral proteins varied depending on the compound and protein target. However, several common residues were identified, including His41, Gln189, Cys145, Thr24, Thr190, Met48, Met165, Ser46, Cys361, Phe392, Ile358, Ser359, Val315, Asn459, Arg349, Ser318, Glu350, Arg457, Pro169, and Arg349. These residues play crucial roles in the structural and functional properties of the viral proteins. The in silico molecular docking results suggest that the selected compounds may potentially inhibit the activity of SARS-CoV-2 proteins, thereby interfering with viral replication. However, further experimental studies are needed to validate these findings and elucidate the precise mechanisms of action of the compounds.

**Table 5:** In Silico molecular docking predictions of compound-protein interactions

Compound	Protein target	Binding site	Binding affinity (kcal/mol)	Key residues
Propolis	Main protease	S1 pocket	−8.04	His41, Gln189, Cys145
Curcumin	Main protease	S2 pocket	−7.29	Thr24, Cys145, Gln189
Quercetin	Main protease	S2 pocket	−6.25	Thr190, Gln189
Ginseng	Main protease	S1 pocket	−10.09	Met48, Met165, Ser46, Gln189, His41
Propolis	Spike protein S1	Receptor binding domain	−8.23	Cys361, Phe392
Curcumin	Spike protein S1	Receptor binding domain	−6.75	Phe392, Tyr393
Quercetin	Spike protein S1	Receptor binding domain	−6.13	Ile358, Ser359
Ginseng	Spike protein S1	Receptor binding domain	−9.57	Leu390, Asn388, Cys361, Cys336, Thr333
Propolis	RNA-directed RNA polymerase	Active site	−8.59	Val315
Curcumin	RNA-directed RNA polymerase	Active site	−8.02	Asn459, Arg349, Ser318
Quercetin	RNA-directed RNA polymerase	Active site	−6.13	Ser318, Asn459
Ginseng	RNA-directed RNA polymerase	Active site	−9.57	Glu350, Arg457, Pro169, Arg349

## Discussion

There is a potential threat to public health associated with SARS-CoV-2, not simply because the virus continues to mutate, reducing the efficacy of vaccines against the zoonoses, but also because new SARS-CoVs may re-emerge, posing a threat to public health (Ahmad 2021). Therefore, it is essential to continue monitoring the virus and to take necessary preventive measures to ensure the public's safety (Allam and Jones 2020). Vaccines and treatments should also be developed and tested to ensure they are effective against potential new SARS-CoVs (Rabaan et al. 2020). A direct target of viral replication has been identified and used in the antiviral studies carried out during the SARS-CoV-2 outbreak (Bhatti et al. 2020). The antiviral RDV acts as a protease inhibitor to inhibit the MPro protein (Bafna et al. 2021). This protein is needed for viral reproduction and is highly preserved among beta Coronaviruses (Slanina et al. 2021). The antiviral drug RDV has demonstrated effectiveness against SARS-CoV-2 infections. It has also been proven effective against other viruses from the beta Coronavirus family (Ko et al. 2020).

The naturally occurring compounds found in natural products are effective against inflammation and viral infections, such as SARS-CoV-2, a newly emerging viral infection. SARS-CoV-2 and host-cell targets resisted various natural compounds and derivatives (Merarchi et al. 2021). This suggested that these natural compounds could be used to develop effective antiviral agents against SARS-CoV-2. Additionally, these compounds could be combined with other antiviral drugs to increase their efficacy (Zhang and Liu 2020). To gain insight into the effectiveness of natural treatments widely used in these regions, this study examined the antiviral activity of four natural compounds used by indigenous people in Tabuk, Saudi Arabia, to combat SARS-CoV-2 infection and COVID-19-related symptoms.

Our results revealed that, at higher concentrations, the compounds displayed no significant cytotoxicity, confirming their safety for in vitro use. A unique aspect of our cytotoxicity experiments is the focus on SARS-CoV-2 infection, where we ensured that the concentrations used were non-toxic but sufficient to inhibit viral replication. We optimized the concentrations to reflect their potential therapeutic ranges, which may differ slightly from conventional cytotoxicity studies due to the viral context. This indicated that these compounds are safe for laboratory studies and possible candidates for further research. Furthermore, these compounds could be used to develop potential therapeutic agents (Zia et al. 2021). Propolis and curcumin, however, were found to be the most safe and viable drugs (Elumalai et al. 2022). The results confirmed those of Sangboonruang et al. (2022), who presented evidence that propolis interferes with the replication of herpes simplex virus type 1 (HSV-1) and type 2 (HSV-2) by targeting viral DNA polymerase.

Furthermore, propolis was reported to significantly diminish HSV-1 and HSV-2 replication, proving that it can be a promising alternative for therapeutic management (Sangboonruang et al. 2022; Magnavacca et al. 2022). SARS-CoV-2 has been shown to interact with several components in propolis (Berretta et al. 2020). For instance, Kumar et al. (2021) presented models showing the interaction between Caffeic Acid Phenethyl Esters (CAPEs) and the SARS-CoV-2 protease. The CAPEs were revealed to block the active site of the SARS-CoV-2 protease, thereby preventing the virus from replicating and spreading (Eberle et al. 2021). Similarly, curcumin can bind to the protease's active site and block the interactions necessary for its enzymatic activity (Omar et al. 2020). As a result, the virus cannot replicate and cause infection. Curcumin also reduces inflammation and pain associated with viral infections.

The primary objective of the antioxidant experiment in our study was to evaluate the antioxidant potential of the selected natural compounds—propolis, curcumin, quercetin, and ginseng—since oxidative stress plays a crucial role in the progression and severity of viral infections, including SARS-CoV-2 (Delgado-Roche and Mesta 2020). The relationship between viral infections and oxidative stress is well-documented, as excessive reactive oxygen species (ROS) production during viral replication can lead to cellular damage, inflammation, and impaired immune responses, exacerbating the severity of infections (Mitran et al. 2021). In the case of SARS-CoV-2, oxidative stress has been implicated in both viral replication and the severe inflammatory responses seen in COVID-19, such as cytokine storms (Rolfo et al. 2022). Therefore, compounds with strong antioxidant properties, like those tested in our study, may reduce oxidative damage and inhibit viral replication indirectly by reducing the cellular environment conducive to viral propagation.

By demonstrating significant antioxidant activity through ABTS, FRAP and CUPRAC assays, we aimed to show that these compounds can neutralize free radicals, which could reduce the severity of COVID-19 symptoms. These antioxidant effects may complement the compounds' direct antiviral activity by targeting viral replication and modulating the excessive immune and inflammatory responses seen in severe COVID-19 cases (Mrityunjaya et al. 2020). Consequently, curcumin has antioxidant properties that protect cells from virus-caused damage (Zahedipour et al. 2020). In this context, quercetin and CAPE both work by interrupting the virus's replication cycle and preventing it from reproducing (Sopjani et al. 2024). As a result of quercetin's inhibition of NF-_k_B, a transcription factor that plays a crucial role in the production of new virus proteins, the virus's protease is inhibited, which is essential for the virus to replicate (Sales-Peres et al. 2023).

Propolis, a natural resinous substance bees produce, has garnered attention for its potential antioxidant properties against COVID-19 (Salatino 2022). Compared to other antioxidants like vitamin C and trolox, propolis demonstrates remarkable efficacy in scavenging free radicals, as evidenced by ABTS, FRAP and CUPRAC assays. This superior scavenging ability suggests propolis could offer heightened protection against oxidative stress, a critical aspect of COVID-19 pathology. Moreover, propolis exhibits dose-dependent effects, significantly enhancing its antioxidant activity as concentrations increase. This dose–response relationship implies that higher concentrations of propolis may yield even more significant benefits in neutralizing free radicals associated with COVID-19 infection (Ripari et al. 2021). Beyond its direct antioxidant effects, propolis contains various bioactive compounds, such as flavonoids, phenolic acids, and terpenes, which possess anti-inflammatory and immunomodulatory properties (Magnavacca et al. 2022; Yosri et al. 2021). These properties could mitigate the inflammatory response and bolster the immune system's defense against COVID-19 (Bhargava et al. 2021).

Furthermore, propolis has demonstrated antiviral activity against various respiratory viruses, including influenza and respiratory syncytial virus (RSV). This antiviral potential suggests that propolis may combat oxidative stress and directly inhibit viral replication, thereby reducing the severity and duration of COVID-19 infection (Asma et al. 2022).

In vitro, our study found that the four selected compounds had antiviral potential versus SARS-CoV-2. The first step consisted of testing how well SARSCoV-2 infected Vero cells to determine the lowest viral concentration required for all replicates to be infected. Our antiviral tests were successful when we used 6.25 µg/mL of each compound. Comparatively to the positive control, the antiviral assay results showed complete inhibition of SARS-CoV-2 using 50–100 g of all compounds. This suggests that the compounds are inhibiting the virus rather than simply preventing it from replicating. This further reinforces the idea that these compounds may be effective antiviral treatments for COVID-19. In our study, propolis proved particularly effective in treating SARS-CoV-2 infection within the early stages and after viral adhesion. This could be explained by propolis containing compounds such as flavonoids and phenolic acids (Kurek-Górecka et al. 2013) that interact with the virus and stop it from replicating (Magnavacca et al. 2022). It also helps to reduce inflammation and stimulate the immune system, which helps fight off the virus (Berretta et al. 2020). According to several studies that have been carried out, propolis inhibits both genome replication and viral assembly in SARS-CoV-2 (Merarchi et al. 2021; Sberna et al. 2022). It should also be noted that SARS-CoV-2 takes advantage of lipid droplets to produce viral membranes and energy (Dias et al. 2020). When you look at these results together, they suggest that propolis might work against viruses in two different ways (Ripari et al. 2021). When propolis was applied in the early stages of an infection, the lack of cytopathic effect (CPE) could mean that it stopped viruses from entering cells or copying themselves. During the post-infection experiments, another possible mechanism could relate to the regulation of lipids since both antiviral mechanisms might be triggered when cells are coated with propolis; nevertheless, additional evidence is crucial to pinpointing the pathway involved (Magnavacca et al. 2022).

Because propolis is used in many medical circumstances, we thought healthy cells would react differently than diseased cells, like bacterial, viral infections, cancer, or inflammatory disease (Zulhendri et al. 2021). As a result, we designed our study to test how propolis (the most effective in vitro antiviral compound in our study) affects Vero cells in healthy and diseased states. We used TNF-β, IL-1β, and IL-10 to cause inflammation in Vero cells to make an in vitro inflammatory disease model. In the next step, we examined the effects of propolis on the cells with and without the addition of cytokines. The data revealed that propolis has an anti-inflammatory effect.

Furthermore, dying tumor cells that undergo immunogenic cell death produce cytokines that modulate immune response. This is because dying tumor cells release molecules called damage-associated molecular patterns (DAMPs) (Land 2020). As a result of these DAMPs, the immune system is alerted to the presence of tumor cells, triggering an immune response that destroys them (Meo et al. 2023). Propolis targets these DAMPs and stimulates the release of TNF-β, IL-1β, and IL-10, which are all cytokines that are crucial for regulating the immune response. Propolis helps activate the immune response and kill the tumor cells by stimulating these cytokines' production. This suggests that propolis has anti-inflammatory properties and highlights the importance of understanding the mechanism behind the anti-inflammatory effects of propolis (Nema et al. 2021).

Interestingly, our compounds significantly inhibited the growth of SARS-CoV-2 based on in vitro compound screening. To gain a greater understanding of the mechanism behind the binding of these active compounds, docking experiments were conducted; however, there were some minor differences in the agreement between docking and in vitro assay results. Accordingly, in the leading protease binding site, curcumin binds to the carboxyl-terminal residues THR24 (H-donor), CYS145 (H-acceptor), and GLN189 (pi-H) with an energy of −7.29 kcal/mol. The interaction between the curcumin and the protease residues is due to its strong binding affinity to the protease's active site, which is stabilized by hydrogen bonds between the curcumin and the CYS145 and THR24 residues and by electrostatic attractions between the curcumin and the GLN189 residue. However, regarding energy value, the bioactive compound of propolis bound effectively with the binding site of the main protease by −8.04 kcal/mol. The propolis was found to have a strong binding affinity with the binding site of the protease, indicating that it may be able to inhibit its activity effectively. The energy value of the propolis was meager, suggesting that it was very stable and unlikely to undergo any spontaneous changes (Sahlan et al. 2021; Hashem 2020).

In addition, quercetin interacted with the main protease's binding site with an energy value of −6.25 kcal/mol by interacting with THR190 (H-donor) and GLN189 (pi-H); this indicates that quercetin binds to the main protease with relatively high affinities, which suggested that it may have the potential to inhibit the enzyme's activity (Petrillo et al. 2022). It was found that the bioactive compound contained in ginseng, ginsenoside re, had a higher energetic requirement (−10.09 kcal/mol) for binding to the main protease binding site than MET48, MET165, SER46, GLN, and HIS41 (pi-H) residues. This proposed that ginsenoside prefers to interact directly with H-donor residues in the binding site of the main protease and that the energy involved in the interaction is lower than with any of the other residues (Xian et al. 2020). Captivatingly, the antiviral potential of the compounds that molecular docking studies have validated could be potential targets for COVID-19 management.

The present study has several limitations that should be considered. First, the sample size for the in vitro experiments was relatively small. This may limit the generalizability of the findings. Second, the study focused solely on in vitro models, and further in vivo studies are necessary to validate the efficacy and safety of the compounds in animal models (Li et al. 2021). Third, while the in silico molecular docking data suggest potential binding interactions between the compounds and the viral proteins, further experimental studies are needed to validate these findings and elucidate the precise mechanisms of action. Functional assays, such as protease inhibition assays, could provide direct evidence of the compounds' ability to interfere with the activity of these viral targets. Larger sample sizes should be used to increase the statistical power and generalizability of the findings. In vivo studies in animal models are necessary to assess the efficacy and safety of the compounds in a more complex biological environment. Additionally, further investigations are needed to elucidate the precise mechanisms of action of the compounds. This could involve biochemical assays, cellular studies, and in vivo experiments. Finally, if preclinical studies continue to show promising results, clinical trials should be conducted to evaluate the safety and efficacy of the compounds in human patients. However, the novel aspects of the study emphasize the comprehensive approach and the inclusion of in silico molecular docking analysis. It also addresses the potential immunomodulatory effects of propolis, which have not been extensively explored in previous studies. Moreover, given the promising docking scores of several individual compounds, future research should also focus on structure–activity relationships (SAR) analysis, which may lead to optimized modifications of these molecules for enhanced bioactivity.

## Conclusion

The current study concluded that the in vitro assay of propolis was the most effective against SARS-CoV-2, with IC_50_ values of 95.89 μg/mL, significantly higher than those of curcumin, quercetin, and ginseng (83.15, 55.23, and 45.43 μg/mL, respectively). Furthermore, the Fa of the compounds decreased even at lower concentrations, further confirming the dose-dependent nature of the compounds' effects on cell viability. This suggests propolis may be the most effective compound of the three tested in this study, with its lowest Fa value and the highest IC_50_ value. This indicates that propolis may be more effective in reducing SARS-CoV-2 activity at lower concentrations than the other two compounds tested, making it a better candidate for further research. Propolis demonstrates superior antioxidant activity compared to curcumin, quercetin, and ginseng, as evidenced by the highest ABTS, FRAP and CUPRAC assay results across all tested concentrations. The results also proposed that propolis and curcumin can remarkably reduce SARS-CoV-2 viral titer. However, quercetin and ginseng are potential candidates for treating SARS-CoV-2 infections, but higher concentrations are needed to achieve maximum efficacy. Further, our data indicated that propolis consumption may effectively reduce the cytokine storm and treat COVID-19. As a result of the molecular docking study, it is demonstrated that the compounds possess a strong binding affinity for the COVID-19 target protein, making them potentially effective antivirals. Accordingly, the compounds bind to viral proteins, preventing them from functioning correctly and hindering virus replication capabilities. These findings collectively contribute to a more comprehensive understanding of these natural compounds' antiviral and immunomodulatory properties against SARS-CoV-2. They also highlight the potential of these compounds as promising candidates for further research and development as antiviral agents.

## Data Availability

The datasets used in the current study are available from the corresponding author on reasonable request.
